# Which factors are associated with adverse prognosis in multiple myeloma patients after surgery? - preliminary establishment and validation of the nomogram

**DOI:** 10.1186/s12957-024-03453-y

**Published:** 2024-06-25

**Authors:** Jun-Peng Liu, Zi-Yu Xu, Yue Wu, Xiang-Jun Shi, Ming Shi, Meng Li, Xin-Ru Du, Xing-Chen Yao

**Affiliations:** 1 Department of Orthopaedic Surgery, Beijing Chaoyang Hospital, Capital Medical University, Beijing, 100020 China; 2 Department of Rheumatology, Beijing Chaoyang Hospital, Capital Medical University, Beijing, 100020 China

**Keywords:** Multiple myeloma, Nomogram, Prognosis, Surgery, Survival analysis

## Abstract

**Background:**

To investigate the prognosis of patients with Multiple Myeloma (MM) after surgery, analyze the risk factors leading to adverse postoperative outcomes, and establish a nomogram.

**Methods:**

Clinical data from 154 patients with MM who underwent surgery at our institution between 2007 and 2019 were retrospectively analyzed. Assessing and comparing patients’ pain levels, quality of life, and functional status before and after surgery (*P* < 0.05) were considered statistically significant. The Kaplan-Meier survival curve was used to estimate the median survival time. Adverse postoperative outcomes were defined as worsened symptoms, lesion recurrence, complication grade ≥ 2, or a postoperative survival period < 1 year. Logistic regression analysis was used to determine the prognostic factors. Based on the logistic regression results, a nomogram predictive model was developed and calibrated.

**Results:**

Postoperative pain was significantly alleviated in patients with MM, and there were significant improvements in the quality of life and functional status (*P* < 0.05). The median postoperative survival was 41 months. Forty-nine patients (31.8%) experienced adverse postoperative outcomes. Multivariate logistic regression analysis identified patient age, duration of MM, International Staging System, preoperative Karnofsky Performance Status, and Hb < 90 g/L as independent factors influencing patient prognosis. Based on these results, a nomogram was constructed, with a C-index of 0.812. The calibration curve demonstrated similarity between the predicted and actual survival curves. Decision curve analysis favored the predictive value of the model at high-risk thresholds from 10% to-69%.

**Conclusion:**

This study developed a nomogram risk prediction model to assist in providing quantifiable assessment indicators for preoperative evaluation of surgical risk.

## Background

Multiple myeloma (MM) is a malignant monoclonal plasma cell proliferative disorder and the second most common hematologic malignancy, accounting for 10% of cases [1]. In recent years, with the continuous improvement of treatment regimens, the survival period of patients with MM has been extended [2, 3], and the 10-year survival rate has reached 30-40% [4]. 80% of patients with newly diagnosed MM have lytic bone lesions, which easily lead to Skeletal-Related Events (SRE) [5], greatly reducing the quality of life of patients. In recent years, with the advancement of surgical techniques and deepening of the concept of combination therapy, the role of surgery in relieving patient pain and improving patient quality of life has become increasingly prominent [6, 7], and the number of MM patients treated with surgery is increasing [8]. Although the prognosis of patients with MM has improved with the continuous improvement of treatment systems, there is still a significant difference in prognosis among patients [9]. Predicting surgical outcomes based on patient characteristics and selecting more suitable treatment regimens are of great significance. Currently, the International Staging System (ISS) is widely used to predict the prognosis of patients with MM. However, ISS only considers the levels of albumin and β2-microglobulin, lacks surgery-related factors, and lacks accuracy in prediction. Patients with the same ISS stage may have significantly different postoperative outcomes. Many studies have supplemented and researched predictive factors beyond ISS; however, most of these study populations are newly diagnosed or undergoing stem cell transplantation, and the evaluated factors are mostly limited to genetic or hematologic markers [10].

Currently, there are no studies describing a predictive model for postoperative outcomes in patients with MM. Utzschneider et al. suggest that, considering the unique nature of MM, surgical evaluations for MM patients should comprehensively consider surgical risks, patient longevity, and quality of life to accurately assess the actual benefits of surgery for each individual patient [11]. The nomogram is a data-integrated visualization tool that quantifies predictive models into probability values, making it convenient for physicians to accurately and simply estimate individual risk [12], which has led to its growing popularity among physicians [13]. The primary aim of this study is to establish a nomogram based on patients’ preoperative data to identify which types of patients are prone to adverse prognoses (e.g., short survival time, symptom exacerbation, recurrence, or complications), thereby assisting physicians in formulating individualized treatment plans and reducing the likelihood of postoperative adverse events [14]. To the best of our knowledge, this is the first study to establish a nomogram predictive model for the postoperative prognosis of patients with MM.

## Patients and methods

This retrospective analysis was conducted on the clinical data of patients with MM who underwent surgery for SRE between January 1, 2007, and December 31, 2019. The surgical plan and timing were jointly determined by experts in orthopedics, hematology, and radiation oncology. This study was approved by the Ethics Committee of Beijing Chaoyang Hospital and was classified as a retrospective audit, exempting the requirement for informed patient consent (Ethics Approval Number: 2018-ke-259).

### Inclusion and exclusion criteria

The inclusion Criteria were as follows: (1) patients diagnosed with MM according to the criteria of the International Myeloma Working Group [15], confirmed by postoperative pathology as myeloma; (2) received surgical treatment; (3) in accordance with ethical requirements, patients aged > 18 years; and (4) expected lifespan ≥ 3 months to ensure sufficient time for surgical benefits. Exclusion Criteria were as follows: (1) active infection at the surgical site or impaired coagulation function (platelets < 80 × 10^9^/L), to reduce the risk of surgical complications; (2) patients with severe comorbidities, infections, or poor physical condition who were not suitable for surgery; and (3) patients with missing data, lost to follow-up, or died due to non-myeloma-related events. During the study period, 7 patients were lost to follow-up, 9 had incomplete electronic records, and 3 died due to non-myeloma-related events. These data were considered completely random losses and were excluded. Ultimately, 154 patients with complete data were included in the statistical analysis.

### Data collection and variables

The demographic characteristics and potential prognostic factors of 154 patients were collected, including general patient information such as age and sex; disease progression details, including duration of MM, Durie-Salmon (DS) stage [16], International Staging System (ISS) stage [17], isotype, anemia status, and preoperative and postoperative chemotherapy; and surgical information such as treated region, number of lesions (number of segments with spinal lesions, number of different sites for non-spinal lesions) and number of prior surgeries (including surgeries for other diseases). Pain severity was assessed using the visual analog scale (VAS) [18], which ranges from 0 (no pain) to 10 (unbearably severe pain), with higher scores indicating greater pain intensity. Quality of life was evaluated using the Karnofsky Performance Status (KPS) [19], which has a total score of 100 points, with higher scores indicating better health status and lower scores indicating poorer health. Patient physical status was assessed using the Eastern Cooperative Oncology Group-performance status (ECOG-PS) [20], which ranges from 0 to 5, with higher scores indicating worse physical and activity status, where 0 represents normal activity and 5 indicates death. These indicators were recorded before and 3 months after surgery. Complications occurring during follow-up, in situ recurrence, and MM-related deaths were recorded. The survival time was measured from the time of surgery to death. Follow-up visits were conducted annually postoperatively, with a minimum follow-up period of 12 months, until the patient’s death or termination of the study. The last follow-up was December 31, 2020. Adverse prognosis was defined as the occurrence of grade 2 or higher complications as defined by Dindo et al. (complications requiring medication or surgery beyond the planned procedure) [21], worsening symptoms, in situ recurrence postoperatively, and survival time < 1 year postoperatively. Patients were classified into poor and good prognosis groups based on their prognosis.

### Statistical analysis

Data were analyzed using IBM SPSS version 25.0 (IBM Corp, Armonk, NY, USA). The Kolmogorov-Smirnov test was used to evaluate the normality of the variables. For normally distributed continuous data, independent sample t-tests were used for between-group comparisons. For skewed distributed continuous and ordinal data, the Mann-Whitney U test was employed for between-group difference analysis. For unordered categorical data, the chi-square test was used, with Pearson Chi-Square selected when the minimum expected count was > 5, Fisher’s Exact Test was used when < 1, and Likelihood Ratio was used for intermediate values for statistical analysis. The Wilcoxon signed-rank test was used to analyze the preoperative and postoperative VAS, KPS, and ECOG-PS scores. All tests were two-sided, and *P* < 0.05 were considered significant. In chi-squared tests, P-values were adjusted using Bonferroni correction. Kaplan-Meier survival curve analysis was used for the survival analysis.

### Establishment of predictive model

Single-factor logistic regression analysis was used to explore the risk factors contributing to adverse prognosis in patients with MM postoperatively. Enter method was employed to enter independent variables of all models. Dummy variables were set for unordered multicategorical data, interaction terms were assessed, and the sample size exceeded ten times the number of the independent variables [22]. Owing to the exploratory nature of this analysis, potential risk factors (*P* < 0.1 were further included in the multivariable logistic regression analysis. Variables with *P* < 0.05 in the multivariable regression analysis were considered independent risk factors, and odds ratios (OR) with 95% confidence intervals (95% CI) were calculated. Correlation tests were conducted to ensure that the correlation coefficient was < 0.5, tolerance was > 0.1, and variance inflation factor was < 5. The Hosmer-Lemeshow goodness-of-fit test was used to validate the calibration of the regression equation. The discrimination of the equation was evaluated using a Receiver Operating Characteristic (ROC) curve. The Area Under the ROC Curve (AUC) was used to assess the predictive performance of continuous variables and ordinal data, with the calculation of cut-off values. The consistency of the unordered categorical data was assessed using the kappa value.

### Establishment and validation of nomogram

The nomogram based on the results of multivariable logistic regression analysis was developed using RStudio (version 4.2.1; RStudio Development Core Team, Vienna, Austria) and the “rms” package.

(1) Calibration: Internal validation was performed using the bootstrap method (1000 times), and a calibration curve was plotted to assess calibration. A 45° calibration curve represents an ideal prognosis prediction.

(2) Discrimination: The ROC curve was plotted, and sensitivity, specificity, concordance index (C-index), and AUC were calculated by bootstrapping method to assess discrimination. C-index and AUC values vary from 0.5 to 1.0, where 0.5 represents random chance and 1.0 indicates a perfect fit. Typically, C-index and AUC values greater than 0.7 suggest a reasonable estimation.

(3) Clinical Benefit: Decision Curve Analysis (DCA) was performed using the R package ‘rmda’ to validate the clinical benefit of the nomogram model.

## Results

### General information

Among the 154 patients, 45 (29.2%) underwent vertebral percutaneous kyphoplasty (PKP) surgery, 41 (26.6%) underwent spinal decompression and internal fixation surgery. Thirty-three (21.4%) patients experienced limb fractures, and 3 (1.9%) patients had rib fractures, for which fracture reduction and fixation combined with bone cement filling were performed. Fourteen (9.1%) patients with soft tissue masses underwent radiofrequency ablation combined with lesion resection surgery. One (0.6%) patient underwent tru-cut biopsy. In 17 (11.0%) patients with lesions in multiple sites, the timing and sequence of treatment were determined through multidisciplinary discussion, with the specific treatment principles for each lesion as mentioned above. The Kolmogorov-Smirnov test indicated that continuous variables such as age and MM duration did not follow a normal distribution. Therefore, statistical analysis was conducted using the Mann-Whitney U test, along with ordinal categorical data. For unordered categorical data such as sex, anemia, and preoperative and postoperative chemotherapy, the Pearson Chi-Square test was used because the minimum Expected Count was > 5. Fisher’s Exact Test was used for isotypes, and surgical site analysis with the minimum Expected Count was < 1. The results showed statistically significant differences between the two groups in terms of age, MM duration, ISS stage, lesion number, preoperative KPS score, and ECOG-PS score (*P* < 0.05, Table 1). The Mann-Whitney U test results revealed significant improvement in VAS, KPS, and ECOG-PS at 3 months postoperatively compared to preoperative values (*P* < 0.05, Table 2), indicating that the majority of patients benefited from surgery.

**Table 1:** The general information of patients

Parameters	Total (*n* = 154)	Good prognosis (*n* = 105)	Poor prognosis (*n* = 49)	*P*
Age (years)	59.33 [11.03]	57.73 [10.71]	62.76 [11.03]	0.009*
Course of the MM (months)	23.23 [20.07]	19.12 [16.39]	35.63 [24.43]	0.001*
Gender	——	——	——	0.569
Male	86 (55.8%)	57 (54.3%)	29 (59.2%)	——
Female	68 (44.2%)	48 (45.7%)	20 (40.8%)	——
ISS stage	——	——	——	0.002*
I	32 (20.8%)	27 (25.7%)	5 (10.2%)	——
II	58 (37.7%)	43 (41.0%)	15 (30.6%)	——
III	64 (41.6%)	35 (33.3%)	29 (59.2%)	——
DS stage	——	——	——	0.873
I	6 (3.9%)	4 (3.8%)	2 (4.1%)	——
II	15 (9.7%)	10 (9.5%)	5 (10.2%)	——
III	133 (86.4%)	91 (86.7%)	42 (85.7%)	——
Isotype	——	——	——	0.528
IgG	82 (53.2%)	58 (55.2%)	24 (49.0%)	——
IgA	40 (26.0%)	28 (26.7%)	12 (24.5%)	——
Light chain	21 (13.6%)	13 (12.4%)	8 (16.3%)	——
Nonsecretory	6 (3.9%)	3 (2.9%)	3 (6.1%)	——
IgD	3 (1.9%)	1 (1.0%)	2 (4.1%)	——
IgM	2 (1.3%)	2 (1.9%)	0	——
Treated region	——	——	——	0.293
Spine (PKP)	45 (29.2%)	34 (32.4%)	11 (22.4%)	——
Spine (open surgery)	41 (26.6%)	25 (23.8%)	16 (32.7%)	——
Limb bones	33 (21.4%)	25 (23.8%)	8 (16.3%)	——
Soft tissue mass	14 (9.1%)	8 (7.6%)	6 (12.2%)	——
Rib	3 (1.9%)	3 (2.9%)	0	——
Surgical biopsy	1 (0.6%)	1 (1.0%)	0	——
Multisite lesions	17 (11.0%)	9 (8.6%)	8 (16.3%)	——
Number of lesions	——	——	——	0.001*
1	91 (59.1%)	71 (67.6%)	20 (40.8%)	——
2	39 (25.3%)	22 (21.0%)	17 (34.7%)	——
3	15 (9.7%)	11 (10.5%)	4 (8.2%)	——
4	7 (4.5%)	1 (1.0%)	6 (12.2%)	——
5	1 (0.6%)	0	1 (2.0%)	——
6	1 (0.6%)	0	1 (2.0%)	——
Number of prior surgeries	——	——	——	0.630
0	85 (55.2%)	58 (55.2%)	27 (55.1%)	——
1	52 (33.8%)	38 (36.2%)	14 (28.6%)	——
2	9 (5.8%)	7 (6.7%)	2 (4.1%)	——
3	2 (1.3%)	0	2 (4.1%)	——
4	3 (1.9%)	1 (1.0%)	2 (4.1%)	——
5	1 (0.6%)	1 (1.0%)	0	——
6	2 (1.3%)	0	2 (4.1%)	——
Preoperative chemotherapy	——	——	——	0.343
Yes	67 (43.5%)	45 (42.9%)	25 (51.0%)	——
No	87 (56.5%)	60 (57.1%)	24 (49.0%)	——
Postoperative chemotherapy	——	——	——	0.253
Yes	101 (65.6%)	72 (68.6%)	29 (59.2%)	——
No	53 (34.4%)	33 (31.4%)	20 (40.8%)	——
Anemia (Hb<90 g/L)	——	——	——	0.082
Yes	60 (39.0%)	36 (34.3%)	24 (49.0%)	——
No	94 (61.0%)	69 (65.7%)	25 (51.0%)	——
Preoperative VAS	8 (7,9)	8 (7,9)	8 (7,9)	0.850
Preoperative KPS	50 (50,60)	60 (50,60)	50 (40,60)	0.007*
Preoperative ECOG-PS	2 (2,3)	2 (2,3)	3 (2,3)	0.001*

Continuous data were represented as x̄ [s], categorical data were presented as n (%), and score-based data were expressed as M (P25, P75)

MM: Multiple Myeloma; ISS: International Staging System; DS: Durie-Salmon; PKP: Percutaneous Kyphoplasty; VAS: Visual Analog Scale; KPS: Karnofsky Performance Status; ECOG-PS: Eastern Cooperative Oncology Group-Performance Status

*P < 0.05

**Table 2:** Comparison of preoperative and postoperative efficacy indicators

Parameters	Preoperative	Three months postoperatively	Z	*P*
VAS	8 (7,9)	3 (2,5)	-10.564	< 0.001*
KPS	50 (50,60)	70 (60,80)	-8.268	< 0.001*
ECOG-PS	2 (2,3)	1 (1,2)	-8.786	< 0.001*

Data were expressed as M (P25, P75)

VAS: Visual Analog Scale; KPS: Karnofsky Performance Status; ECOG-PS: Eastern Cooperative Oncology Group Performance Status

*P < 0.05

### Typical cases

**Fig. 1:**
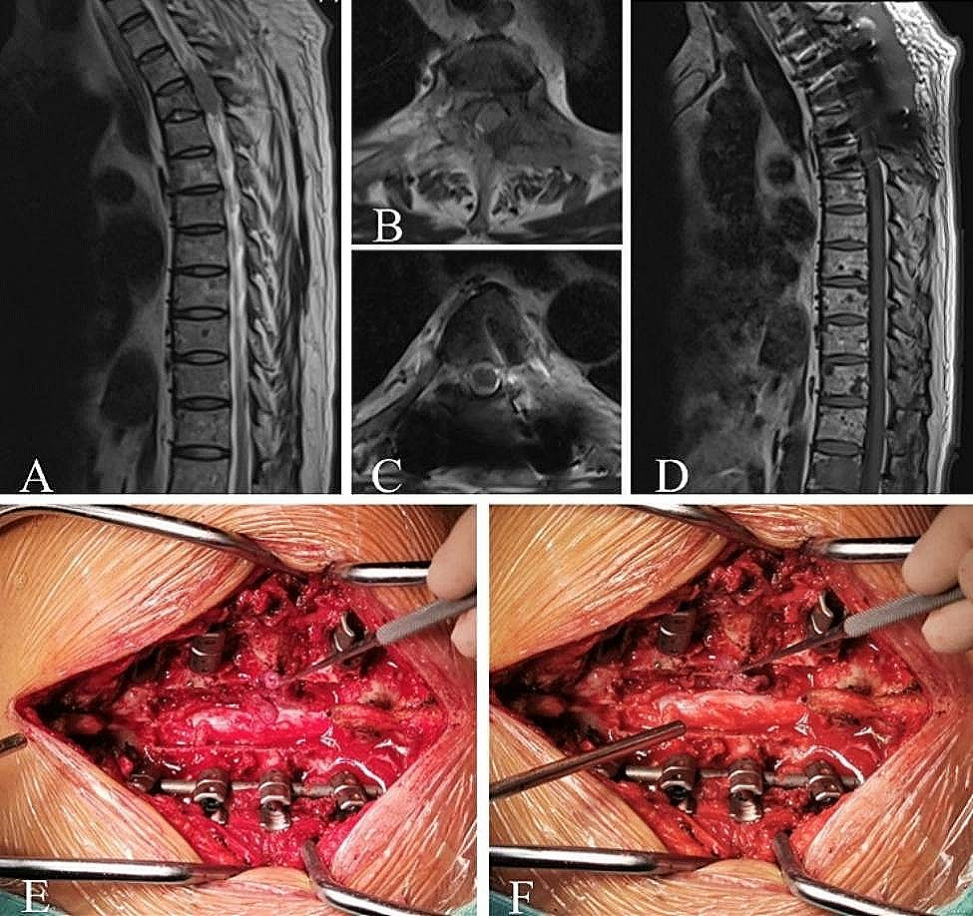
Spinal cord compression due to plasmacytoma. **(A-B)** Preoperative MRI shows a tumor compressing the spinal cord at the T2-4 levels, with epidural spinal cord compression graded as level 3. **(C-D)** Postoperative MRI reveals cerebrospinal fluid filling around the spinal cord without obvious compression, with pedicle screws well-placed and spinal sequences stable. **(E-F)** Intraoperatively, fish-like tumor tissue is visible, and laminectomy and tumor resection are performed to decompress the spinal cord

Typical Case 1 (Spinal Cord Compression, Fig. 1): A 55-year-old male diagnosed with MM for 7 years. DS stage III, ISS stage I. During chemotherapy, a sudden loss of sensation below the nipples and bilateral lower limb paralysis occurred. Three days later, the patient was transferred to the orthopedic department with loss of sensation below the nipples, bilateral lower limb muscle strength grade 0, and bilateral pathological signs (+). Magnetic Resonance Imaging (MRI) revealed an intraspinal mass at the T2-4 level. The procedure involved posterior decompressive laminectomy from T1 to T5, tumor resection, radiofrequency ablation, pedicle screw fixation, and T3 bone cement filling. Three weeks postoperatively, sensation improved compared to before surgery, with the sensory plane descending to the level of both knees, and muscle strength at grade 1 in both lower limbs. One year postoperatively, the sensation recovered, with grade 4 muscle strength in the left lower limb and grade 3 muscle strength in the right lower limb.

**Fig. 2:**
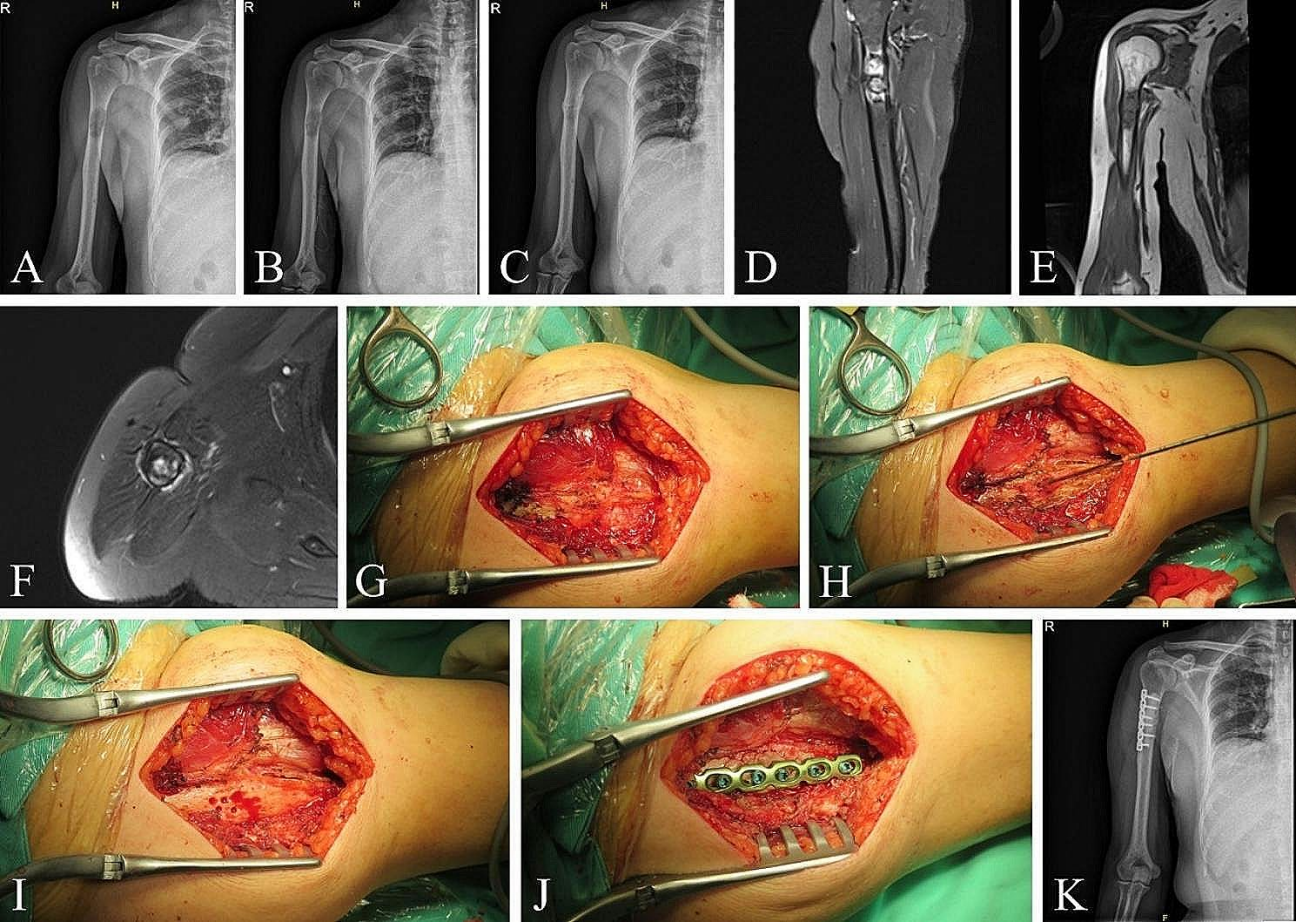
Right humeral pathological fracture in the upper segment. X-rays taken 5 months **(A)**, 4 months **(B)**, and 1 week **(C)** preoperatively show local thinning of the bone cortex and discontinuity of part of the bone cortex. **(D-F)** MRI taken 3 months preoperatively shows a soft tissue mass, approximately 2.0 × 1.9 × 4.8 cm, with unclear boundaries. **(G)** Exposure of the fracture and reveals callus formation. **(H)** Radiofrequency ablation. **(I)** Bone drilling, opening of bone window, and removal of tumor from the marrow cavity. **(J)** Bone cement filling, fixation with plate and screws. **(K)** X-ray taken 3 days postoperatively

Typical Case 2 (Pathological Fracture of Limbs, Fig. 2): A 54-year-old male diagnosed with MM for 6 years, DS stage III, ISS stage I. He had been experiencing continuous pain in the right upper arm for six months. MRI revealed a mass in the upper segment of the right humerus. Radiographs showed thinning of the cortical bone with discontinuities. He underwent four cycles of V-DECP chemotherapy, with slight relief of pain, but still required oral opioid analgesics, with a VAS score of 6. The patient underwent tumor resection, radiofrequency ablation, internal fixation, and bone cement filling. Two weeks postoperatively, the patient’s pain significantly decreased, with VAS reduced to 2 X-rays indicated good alignment of the fracture, and after suture removal, the patient began the next stage of chemotherapy.

### Complications

**Fig. 3:**
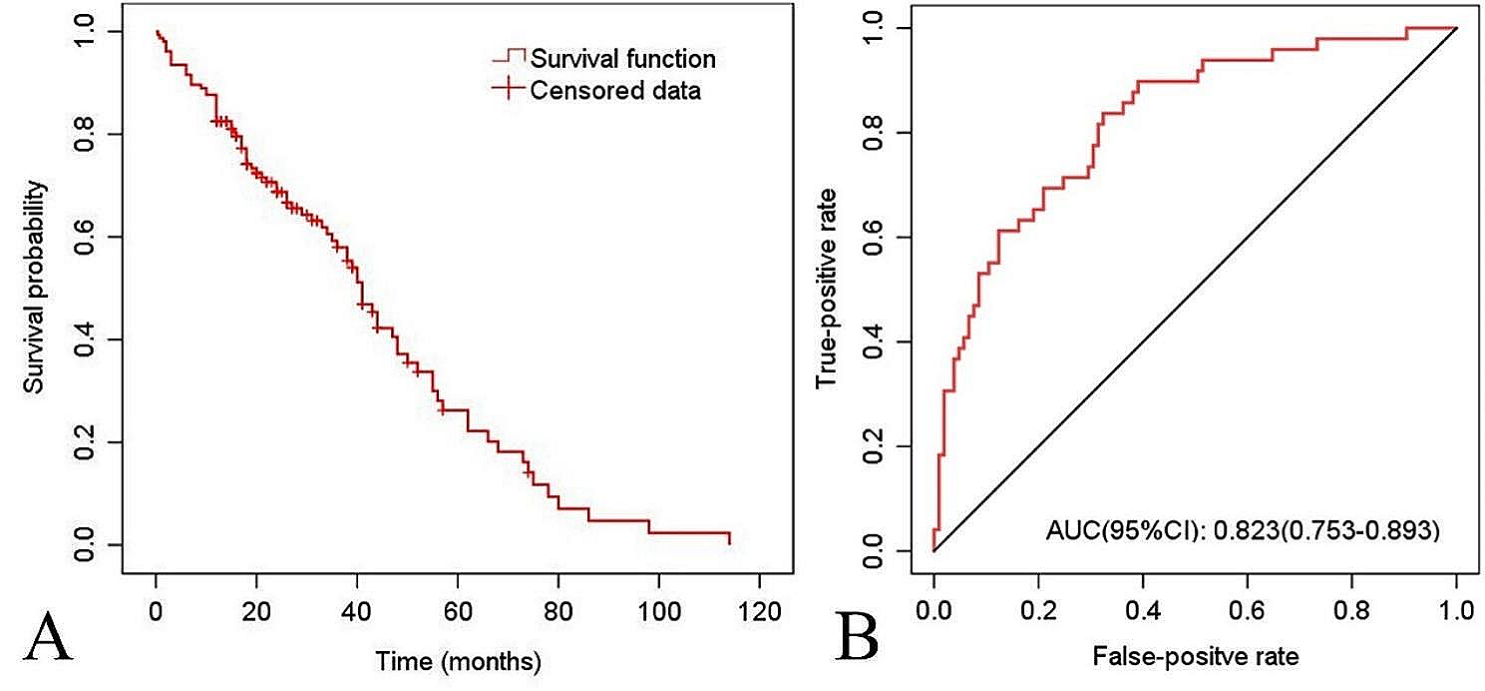
**(A)** Kaplan-Meier survival curve showing 12-month survival was 82.5%, and Kaplan-Meier estimate of median overall survival for all patients was 41.00 months. **(B)** Receiver operating characteristic curve resulting from the multivariate logistic regression. AUC, area under the curve

Fifteen patients (9.7%) experienced postoperative in-situ recurrence or worsening of symptoms. Twenty-one patients (13.6%) had grade ≥ 2 complications (Table 3). Twenty-seven patients (17.5%) had a postoperative survival period of < 1 year. A total of 49 patients (31.8%) had poor prognosis as defined in the article. By the end of the study, 85 patients (55.2%) succumbed to MM-related mortality, with postoperative survival times ranging from 0.35 to 114.00 months, and a median postoperative survival time of 41.00 months (Fig. 3A).

**Table 3:** Compilation of complications (*n* = 21)

Complications	*n* = 21(100%)
Pulmonary infection	8 (38.1%)
Surgical site infections	3 (14.3%)
Cerebral infarction	2 (9.5%)
Bone cement leakage	2 (9.5%)
Nerve compression	2 (9.5%)
Internal fixation loosening	1 (4.8%)
Urinary infections	1 (4.8%)
Haemothorax	1 (4.8%)
Gastrointestinal bleeding	1 (4.8%)

Data were presented as n (%). Listed complications were ≥ grade 2, and wound infections managed solely in the ward were excluded

### Regression analysis

Single-factor logistic regression analysis showed that age, duration of MM, ISS stage, number of lesions, Hb < 90 g/L, preoperative KPS score, and preoperative ECOG-PS score were correlated with patient prognosis (*P* < 0.1, Table 4). Spearman correlation analysis revealed a strong correlation between the ECOG-PS and KPS (*r* =-0.838, *P* = 0.000). Due to the broader range and better representativeness of the KPS, the ECOG-PS was excluded. These potential influencing factors were included in the multivariate logistic regression analysis. Ultimately, age, duration of MM, ISS stage, preoperative KPS score, and Hb < 90 g/L were identified as independent influencing factors leading to poor postoperative prognosis (*P* < 0.05, Table 5). The AUC and cut-off values for each parameter were calculated. Finally, age ≥ 64 years, MM duration ≥ 35 months, ISS stage III, preoperative KPS score ≤ 40 points, and number of lesions ≥ 2 were identified as independent risk factors for poor prognosis. A consistency test for Hb levels < 90 g/L resulted in a kappa value of 0.139. The sensitivity of the test were 0.49, and the specificity was 0.66. The AUC (95% CI) of the multivariate logistic regression analysis was 0.823 (0.753–0.893), with a *P*-value of 0.000, indicating a higher predictive accuracy than that of single-factor regression analysis (Fig. 3B). The omnibus *P* value was 0.000, indicating that compared to the null model, this model has better clinical effectiveness. The − 2 Log Likelihood was 145.038, Cox & Snell R^2^ was 0.266, Nagelkerke R^2^ was 0.373. The Hosmer-Lemeshow statistic was 3.827 with a p-value of 0.872, suggesting a good fit of the regression curve.

**Table 4:** Results from univariate logistic regression

Parameters	OR	95%CI	*P*
Age (years)	1.046	(1.011–1.082)	0.010*
Course of the MM (months)	1.031	(1.013–1.051)	0.001*
Gender	0.819	(0.412–1.628)	0.569
ISS stage	2.183	(1.323–3.603)	0.002*
DS stage	0.947	(0.466–1.927)	0.881
Isotype	——	——	0.707
Treated region	——	——	0.501
Number of lesions	1.956	(1.335–2.866)	0.001*
Number of prior surgeries	1.291	(0.951–1.752)	0.101
Preoperative chemotherapy	1.389	(0.703–2.743)	0.344
Postoperative chemotherapy	0.665	(0.329–1.342)	0.255
Anemia (Hb<90 g/L)	1.840	(0.923–3.668)	0.083*
Preoperative VAS	1.040	(0.853–1.269)	0.696
Preoperative KPS	0.967	(0.944–0.990)	0.006*
Preoperative ECOG-PS	2.056	(1.332–3.172)	0.001*

The isotype and treated region were unordered multicategorical variables, and the dummy variables were set

MM: Multiple Myeloma; ISS: International Staging System; DS: Durie-Salmon; VAS: Visual Analog Scale; KPS: Karnofsky Performance Status; ECOG-PS: Eastern Cooperative Oncology Group Performance Status

*P < 0.1

**Table 5:** Results from multivariable logistic regression analysis and ROC

Parameters	Multivariate logistic regression			ROC			
	OR	95%CI	*P*	AUC	95%CI	*P*	Cut-off
Age (years)	1.041	(1.002–1.081)	0.039*	0.632	(0.536–0.727)	0.009*	63.5
Course of the MM (months)	1.037	(1.013–1.060)	0.002*	0.674	(0.579–0.769)	0.001*	35.0
ISS stage	2.360	(1.285–4.332)	0.006*	0.648	(0.556–0.740)	0.003*	2.5
Preoperative KPS	0.969	(0.939–0.999)	0.044*	0.631	(0.273–0.466)	0.009*	45.0
Anemia (Hb<90 g/L)	2.629	(1.140–6.062)	0.023*	——	——	——	——
Number of lesions	1.598	(0.990–2.578)	0.055	0.648	(0.552–0.744)	0.003*	1.5

Preoperative KPS is a protective factor for poor prognosis, with the AUC representing the area under the ROC curve after inversion. The cut-off value corresponds to the point at which the Youden index is maximized

ROC: Receiver Operating Characteristic; AUC: Area Under the Curve; MM: Multiple Myeloma; ISS: International Staging System; KPS: Karnofsky Performance Status

*P < 0.05

### Nomogram

**Fig. 4:**
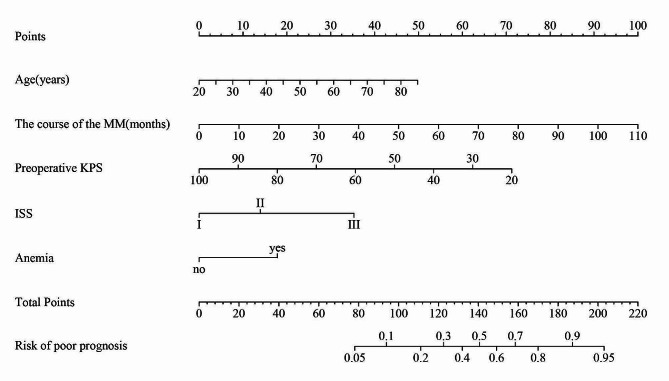
Nomogram for predicting individual prognosis based on data from 154 MM patients underwent surgery. Anemia is defined as Hb < 90 g/L

A nomogram (Fig. 4) was developed based on the five independent influencing factors selected from the regression analysis. Each predictor was assigned a score. The patient’s total score was calculated by summing the scores of each variable. By plotting a vertical line corresponding to the total score on the x-axis below, the probability of the patient experiencing the adverse outcome defined in the text can be obtained.

**Fig. 5:**
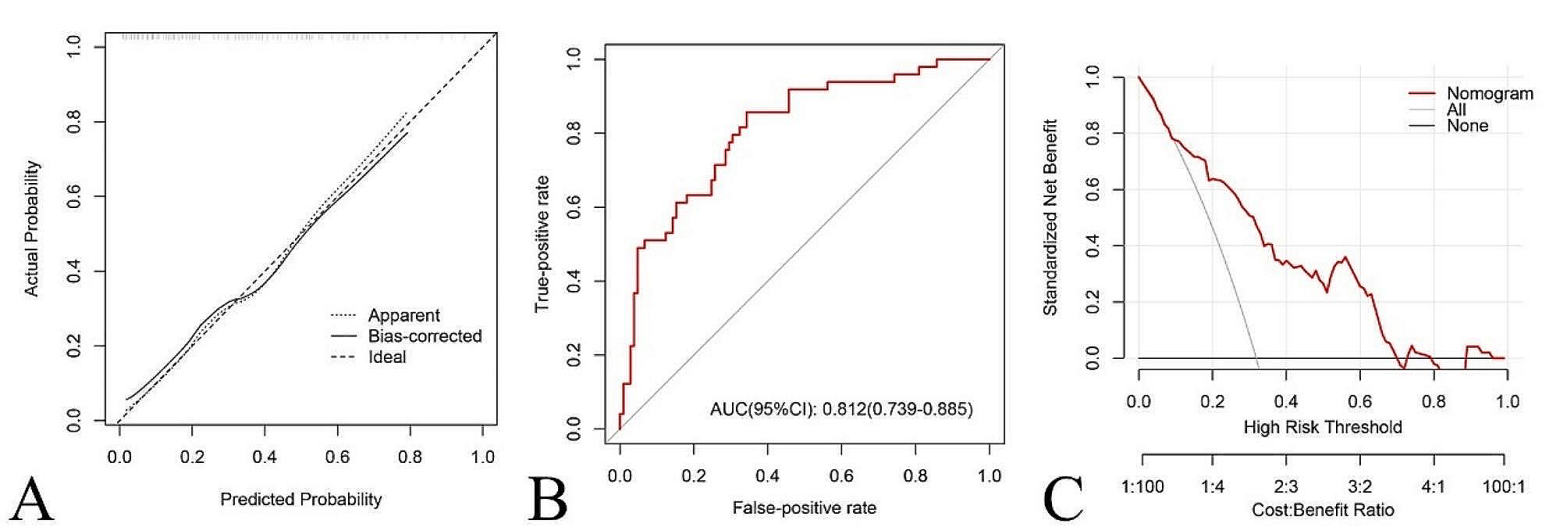
**(A)** The calibration curve of the nomogram model. **(B)** Receiver operating characteristic curve to assess predictive accuracy of the nomogram model. AUC, area under the curve. **(C)** Decision curve analysis of the nomogram model

In the calibration curve of the nomogram, the x-axis represents the predicted probability of adverse outcomes by the nomogram, while the y-axis represents the actual probability of adverse outcomes. The ideal curve for a perfect predictive model corresponds to a 45° diagonal line. The apparent curve represents the actual information of the entire cohort (*N* = 154), while the bias-corrected curve is obtained through bootstrapping (B = 1000 repetitions) to correct any bias. The ideal and apparent lines were very close in the calibration curve (Fig. 5A), with a mean absolute error of 0.02, and the mean squared error was 0.00045, indicating the high consistency of the model. The C-index of the nomogram was 0.812 which was > 0.7 (95% CI: 0.739–0.885, Fig. 5B), with a predicted sensitivity of 0.857 (95% CI: 0.759–0.955) and a predicted specificity of 0.657 (95% CI: 0.566–0.748), demonstrating good discrimination. In the DCA, the y-axis represented standardized net benefit, with the first x-axis indicating the high-risk threshold and the second x-axis indicating the cost: benefit ratio. The grey horizontal line represented “none”, indicating no intervention with a net benefit of 0 for all patients, while the sloping line represented “all”, indicating intervention for all samples, with a net benefit curve having a negative slope. Within the threshold range of 10–69%, the model curve was positioned above both the “none” and “all” lines, indicating a net benefit > 0, signifying the model’s effective clinical guidance in this range (Fig. 5C).

## Discussion

SRE refers to a series of clinical complications resulting from osteolytic destruction caused by myeloma, including pathological fractures, spinal cord and nerve compressions, osteoporosis, hypercalcemia, and bone pain, with an incidence rate of > 80% [5]. These complications significantly diminish the quality of life of the patients. Hematological defects [20], poor bone quality, and immunosuppressive effects of chemotherapy collectively increase the risk of surgical complications in patients [8, 23], thereby limiting the application of surgery. In recent years, advancements in chemotherapy drugs and regimens have improved patients’ bone conditions. Continuous improvements in surgical techniques and instruments have led to improved control of surgical trauma and complications. These factors increase the feasibility of SREs surgery.

The majority of patients undergoing surgery experience pathological fractures in the spine or limbs, or spinal cord compression, accounting for 77.3% of the patients in this study. For unstable limb bone lesions, surgery is significantly superior to radiotherapy or chemotherapy in relieving pain, restoring bone stability, and improving limb function [24]. However, controversy remains regarding the treatment of spinal lesions using radiation therapy or surgery. The International Myeloma Working Group recommends the use of PKP in patients with vertebral fractures that are accompanied by refractory pain. Patients undergoing spinal open surgery in this study had previously undergone non-surgical treatments, led by hematologists, including conventional external beam radiation therapy, but did not achieve the desired effects. Additionally, patients with ESCC grade ≥ 2 face a risk of irreversible damage to the spinal cord and nerves because the spinal cord is tightly surrounded by tumors when high-dose stereotactic radiation therapy is employed. Although immune deficiency associated with MM may increase the risk of complications [25], and a longer postoperative recovery period may lead to delays in subsequent treatment. Following multidisciplinary assessment at our institution, surgery is deemed necessary under these circumstances. Surprisingly, patients obtained exceeded expectations following these “last resort” surgeries. The VAS, KPS, and ECOG-PS scores improved significantly postoperatively compared with the preoperative scores. Surgery rapidly and effectively relieved patient pain, improved their physical condition, and enhanced their quality of life [7, 26]. Twenty-one patients (13.6%) experienced grade 2 or higher surgical complications. To date, there is no consensus on the occurrence of surgical complications in MM patients, and different studies have reported significantly different complication rates, ranging from 3.9 to 35% [6, 8, 27]. MM can cause hypogammaglobulinemia, T cell dysfunction, and granulocytopenia, leading to a higher risk of postoperative infection [25]. The most common postoperative complication in this group of patients is pulmonary infection, consistent with previous research findings [27, 28]. However, surprisingly, few patients experienced incision-related complications such as incision infection or non-healing. The few cases of incision infection that required treatment were all patients who prematurely proceeded to the next stage of postoperative treatment. The treatment plan and timing of surgery for this group of patients were jointly determined by a multidisciplinary team; in most cases, there was sufficient time for patients to recover before proceeding to the next stage of treatment. Multidisciplinary joint management and comprehensive treatment planning are crucial to reduce complications. During treatment, we found that the expression of VEGF, CD3, and CD34 at the incision edge of MM patients was high, indicating rich capillaries and a good blood supply, which may be one of the reasons why incisions in MM patients healed smoothly. Fifteen cases (9.7%) of patients experienced worsening symptoms or local recurrence postoperatively, indicating a high postoperative recurrence rate of SREs [6, 8]. Early postoperative radiotherapy or chemotherapy can limit disease progression and reduce the postoperative recurrence rate [29, 30]. Despite the majority of patients receiving standardized treatment postoperatively, as SRE patients undergoing surgery are generally in the late stages of the disease and often have end-stage organ failure [31], the one-year postoperative mortality rate for patients was 17.5%, and the median postoperative survival time was 41 months. A study based on the SEER database indicates that the average survival time for patients undergoing surgery for MM is 58 months [32]. Considering the previously reported one-year postoperative mortality rate ranging from 9.1–27.4% [33–35], and the median survival period ranging from 19 to 79 months [33, 36], it is evident that postoperative survival periods in patients vary greatly. Although updates to myeloma drugs and treatment regimens may prolong patient survival, the substantial variation in prognosis cannot be solely explained. Other characteristics associated with prognosis may explain this difference.

Despite the description of numerous genetic or hematological prognostic markers, postoperative analysis specific to MM patients remains lacking, and surgery-related factors explaining the heterogeneity in postoperative prognosis of MM patients have yet to be elucidated. Surgical treatment of SRE still lacks reference clinical data. DS staging can reflect tumor burden; however, most patients with MM requiring surgery are in the DS-III stage, which has poor discrimination for patients. ISS staging has a reference value for patient survival outcomes [37–39], is currently widely used as a prognostic assessment system. R-ISS adds lactate dehydrogenase and genetic indicators to ISS but is rarely applied in clinical practice [10]. The results of this study affirm the predictive value of ISS staging for patient prognosis. However, ISS staging only considers two indicators, albumin and β2-microglobulin, which limits its predictive power. Patients with the same ISS stage often exhibit different prognoses, which can be attributed to other patient characteristics. Previous studies have proposed potential prognostic factors for newly diagnosed MM patients or those treated with chemotherapy or autologous stem cell transplantation. This study comprehensively considered these factors and incorporated them with ISS staging and other significant surgery-related characteristics into the predictive system to enhance the model’s predictive capability. Through regression analysis, age, MM duration, ISS stage, preoperative KPS score, and Hb level < 90 g/L were identified as independent influencing factors and included in the nomogram model. Based on our patient data, the AUC of the ISS was 0.648, while that of the nomogram model was 0.812. Generally, a larger AUC indicates higher model discrimination, with an AUC > 0.7 suggesting strong predictive performance [40].The model performed well in calibration, discrimination, clinical utility, and prediction, providing good predictive value. In newly diagnosed MM patients, age, disease duration, and β2-MG are often used as indicators for predicting patient survival [41]. These indicators are also applicable in extramedullary infiltration of myeloma [40] and in our patient cohort. This suggests that patients undergoing surgery for myeloma bone disease still follow prognostic patterns of general MM patients. Additionally, this study found that hemoglobin levels and ECOG scores were related to patient prognosis. These prognostic factors have been mentioned in only a limited number of articles, such as prognostic model studies involving large number of cases [42] or patients with extramedullary disease [14]. The differences in influencing factors could be attributed to variations in patient characteristics or the small scale of cases in most current literature. As the sample size increases, the prognostic factors may be updated.

The results of this study indicated that age was an independent risk factor affecting postoperative prognosis, consistent with the findings of Lee et al. [43]. This phenomenon has been observed not only in MM patients but also in patients with bone metastases from lung adenocarcinoma [44] and non-metastatic high-grade limb osteosarcoma [45]. Furthermore, a cutoff age of 64 years was identified, which is similar to the findings of Lakshman et al., who determined that an age greater than 65 years is a risk factor for poor prognosis in MM patients with t(11;14) based on a study of 372 cases [46]. Elderly patients often have multiple comorbidities, slower postoperative recovery, and higher perioperative risks [27]. Additionally, owing to the foreseeable shorter life expectancy and higher treatment risks, treatment plans for elderly patients are often conservative. Conversely, younger patients can tolerate the side effects of chemotherapy and benefit from therapy, leading to improved survival rates with active postoperative adjuvant therapy. As the disease progresses, the tumor burden gradually increases, and the duration is emerging as an independent risk factor for adverse prognosis in MM patients, especially those with durations exceeding 35 months who are more prone to adverse outcomes following surgery. Anemia is a typical manifestation in MM patients, with 70% presenting at diagnosis and 97% developing it during disease progression [47]. MM-associated anemia is caused by various mechanisms, including cytokine-induced apoptosis of red blood cells secreted by myeloma cells, erythroblastic island destruction leading to decreased red blood cell production, insufficient erythropoietin production due to renal impairment, impaired response to erythropoietin due to marrow replacement by plasma cells, and impaired utilization of stored iron [48–50]. Therefore, anemia is a consequence of proliferative and active myeloma cells, reflecting to some extent the progression of myeloma and kidney damage [51], and is considered an important indicator of end-organ failure in patients with MM [52]. Considering the widespread prevalence of anemia in MM patients, this study utilized Hb < 90 g/L (moderate/severe anemia) to evaluate patient prognosis [53], in order to enhance discrimination. Preoperatively, 60 patients (39%) had Hb levels < 90 g/L. Multifactorial regression analysis suggested Hb levels < 90 g/L as an independent influencing factor for patient prognosis. Anemia is significantly associated with increased perioperative adverse events, not only in MM but also in other diseases [54], possibly due to tissue ischemia and hypoxia. This emphasizes the necessity for identifying the causes of anemia preoperatively and addressing it [51]. Regression analysis results demonstrated that preoperative KPS and ECOG-PS scores were factors influencing patient prognosis. It is evident that postoperative records better reflect patient prognosis [55]. However, considering that the purpose of this study was to provide references for preoperative decision-making, the KPS and ECOG-PS values included preoperative records, unavoidably reducing the predictive accuracy of these parameters. Nonetheless, KPS showed meaningful statistical results. The lower the KPS and the higher the ECOG-PS score, the worse the prognosis of the patients [27, 55]. Currently, ECOG has been proven to be associated with an increased risk of infection in patients with MM and has been incorporated into the infection risk prediction models for MM patients, such as the FIRST and GEM-PETHEMA models [56]. When the preoperative KPS score is ≤ 40 (indicating that patients are unable to perform activities of daily living or worse, often due to spinal lesions or comorbidities such as cerebral infarction or spinal cord injury), the prognosis is poor. A multicenter study based on data from palliative care for MM patients identified the optimal KPS threshold for predicting patient mortality to be 30 [19], which is close to the results of this study. Utilizing a nomogram to comprehensively assess whether the benefits of surgery outweigh the risks is a prerequisite for surgical intervention. As a clinical decision support tool, the nomogram can clearly illustrate the weight of specific predictive factors affecting the postoperative prognosis of myeloma patients. It provides a scientific and intuitive assessment of surgical risks, identifies patient subgroups that need attention, and helps formulate treatment plans to reduce adverse events. Special attention should be given to elderly patients, those with a long disease course of MM, or those with poor functional status, and a comprehensive evaluation of their physical condition is essential. Furthermore, due to the aggressiveness of the disease, preoperative functional assessments of the heart, liver, and kidneys are crucial. Surgical intervention in patients with long periods of spinal cord compression results in limited symptom improvement [7]; therefore, treatment plans for such patients need to be formulated with caution.

The number of lesions is a potential prognostic factor for patients with MM. Compared with patients with a single lesion, those with two or more lesions generally exhibit poorer outcomes following surgery. The presence of multiple lesions suggests a larger tumor burden, closer disease progression to the advanced stage, and consequently, a worse expected survival outcome [7, 25, 26]. However, no prognostic differences were found based on the lesion location. On one hand, MM being a non-solid tumor may diminish the predictive accuracy of conventional indicators commonly used for solid tumors, such as tumor diameter and distant metastatic locations. On the other hand, surgical complications following soft tissue lesions are uncommon because they do not involve bone repair or reconstruction. However, when lesions present as soft tissue, it often indicates that tumor cells have undergone immune escape and acquired the ability to survive outside the bone marrow microenvironment, leading to shorter survival [57]. As this study integrates survival, complications, exacerbation, and recurrence, defining all as poor prognosis, while enhancing the comprehensive analysis of adverse outcomes, it may offset the differences in patient prognosis caused by different lesion locations.

A total of 67 patients (43.5%) received chemotherapy before surgery, and 101 patients (65.6%) received chemotherapy after surgery. Some scholars argue that preoperative chemotherapy may compromise patient immune function, leading to an increased risk of complications under the dual impact of surgery and chemotherapy [8, 58]. Additionally, individual studies [28] have observed that immediate postoperative chemotherapy or radiotherapy may increase the risk of implant failure. However, other scholars believe that chemotherapy or radiotherapy can eradicate residual tumor cells from surgery and improve overall patient survival [34, 57]. Both preoperative and postoperative chemotherapy did not show a difference in patient prognosis. On one hand, the different combinations of chemotherapy, surgery, and radiotherapy may have varying effects on the prognosis of patients. On the other hand, the timing of chemotherapy, whether administered one week or one month after surgery, may result in drastically different outcomes. As our institution lacks radiotherapy equipment, subsequent radiotherapy for patients was conducted at other institutions, resulting in the absence of radiotherapy records; thus, radiotherapy data were not included.

There are still some shortcomings in this study. First, some patients had already experienced clinical remission, without differentiation from other patients, which may have led to longer recorded disease durations than actual illness durations. Second, the formulation of patient surgical plans and certain evaluation criteria is subjective and, inevitably introduces confounding factors. Additionally, limited by its retrospective design, the study carries inherent potential biases. Third, this study did not assign weights to different adverse prognostic outcomes, did not analyze patient characteristics with multiple concurrent adverse prognostic outcomes, and included a limited number of factors, thereby failing to fully reflect some significant influencing factors. Fourth, due to the small sample size and the loss of data for some patients, external validation and high-confidence subgroup analyses were not performed. Finally, MM incidence, disease progression, and response to treatment vary significantly among different racial groups [59]. However, this involves more complex multicenter studies and larger sample sizes; thus, this study did not analyze racial factors, limiting the extrapolation of this model. Investigating the relationship between race, social factors, other serum biomarker assessments, surgical-related indicators, genetic phenotypes, and patient prognosis is crucial for future research directions. Utilizing existing cases to build models based on deeper computer algorithms holds important implications [60]. Additionally, investigating the mechanisms behind influencing factors deserves attention.

## Conclusions

The model is the first nomogram developed specifically for assessing surgical prognosis in MM patients, utilizing novel efficacy assessment criteria, with good efficacy validation. It can assist surgeons in evaluating surgical risks, aiding clinical decision-making, and postoperative management.

## Data Availability

No datasets were generated or analysed during the current study.

## Abbreviations

MM: Multiple Myeloma
SRE: Skeletal-Related Events
ISS: International Staging System
PKP: Percutaneous Kyphoplasty
DS: Durie-Salmon
VAS: Visual Analogue Scale
KPS: Karnofsky Performance Status
ECOG-PS: Eastern Cooperative Oncology Group-performance status
ROC: Receiver Operating Characteristic
AUC: The Area Under the ROC Curve
DCA: Decision Curve Analysis
MRI: Magnetic Resonance Imaging
